# Vitamin C levels in patients with SARS-CoV-2-associated acute respiratory distress syndrome

**DOI:** 10.1186/s13054-020-03249-y

**Published:** 2020-08-26

**Authors:** Luis Chiscano-Camón, Juan Carlos Ruiz-Rodriguez, Adolf Ruiz-Sanmartin, Oriol Roca, Ricard Ferrer

**Affiliations:** 1 Intensive Care Department, Vall d’Hebron Hospital Universitari, Vall d’Hebron Barcelona Hospital Campus, Passeig Vall d’Hebron 119-129, Barcelona, 08035 Spain; 2 Shock, Organ Dysfunction and Resuscitation Research Group, Vall d’Hebron Hospital Universitari, Vall d’Hebron Barcelona Hospital Campus, Passeig Vall d’Hebron 119-129, Barcelona, 08035 Spain; 3 Departament de Medicina, Universitat Autònoma de Barcelona, Bellaterra, 08193 Spain; 4 Ciber Enfermedades Respiratorias (Ciberes), Instituto de Salud Carlos III, Madrid, Spain

Vitamin C is an antioxidant with anti-inflammatory and immune-supportive properties. Its levels are decreased in patients with sepsis-related acute respiratory distress syndrome (ARDS). Moreover, a significant number of patients with severe acute respiratory syndrome coronavirus-2 (SARS-CoV-2) disease developed ARDS [1]. Therefore, we hypothesized that ARDS coronavirus disease 2019 (COVID-19) patients may present vitamin C deficiency.

Plasma vitamin C levels in a population of adult ICU patients COVID-19 who met ARDS criteria according to the Berlin definition [2] were prospectively measured. The study was approved by the local Clinical Research Ethics Committee (PR (AG)270/2020). Main characteristics of the population included are presented in Table 1. None of the patients included presented shock or sepsis on admission. Equally, no bacterial co-infection during their ICU course was documented. All patients survived. Vitamin C was determined by high-performance liquid chromatography with photodiode detector (detection limit 1.5 mg/L). Vitamin C reference values in general population used to be above 5 mg/L. Seventeen patients (94.4%) had undetectable vitamin C levels and 1 patient had low levels (2.4 mg/L).

**Table 1:** Clinical characteristics of the COVID-19 patients included. We have included the worst PF and highest PEEP. Results of continuous variables are expressed as mean and standard deviation or median and interquartile range as appropriate. Categorical variables are expressed as frequency (percentage). *SOFA* sequential organ failure assessment, *APACHE II* Acute Physiology and Chronic Health disease Classification System II, *ICU* intensive care unit, *PF* PaO2/FiO2 ratio, *PEEP* positive end-expiratory pressure, *AKI* acute kidney injury, *CRRT* continuous renal replacement therapy, *LMWH* low-molecular-weight heparin

Clinical characteristics	COVID-19 ARDS (*n* = 18)
Age (mean, standard deviation, years)	59 ± 9
Male (*n*, %)	7 (38)
SOFA score (median, interquartile range, points)	4 (1)
APACHE II score (mean, standard deviation, points)	16.2 ± 1.6
Interval between ICU admission and blood samples extraction for vitamin C measurement (mean, standard deviation, days)	17.5 ± 1.7
Interval between intubation and blood samples extraction for vitamin C measurement (mean, standard deviation, days)	17.5 ± 1.7
ARDS-related variables	
PaO_2_/F_I_O_2_ at the time of vitamin C measurement (mean, standard deviation, mmHg)	94.4 ± 5.9
PEEP (cmH_2_O) at the time of vitamin C measurement (median, interquartile range, points)	13.6 (3)
Neuromuscular blockade during ICU admission (*n*, %)	18 (100)
Prone position during ICU admission (*n*, %)	17 (94)
Renal failure	
AKI (*n*, %)	3/18 (16)
AKI I (*n*, %)	2/3 (66)
AKI III (*n*, %)	1/3 (33)
CRRT (*n*, %)	1/18 (6)
COVID-19-related therapies	
Antivirals (*n*, %)	14 (77)
Hydroxychloroquine (*n*, %)	17 (94)
Tocilizumab (*n*, %)	13 (72)
Methylprednisolone (*n*, %)	10 (55)
LMWH anticoagulant (*n*, %)	8 (44)
Outcomes	
Length of ICU stay (mean, standard deviation, days)	28.4 ± 3.4
Number of hospital survivors (*n*, %)	18 (100)

To our knowledge, this is the first study to analyze the levels of vitamin C in patients with SARS-CoV-2-associated ARDS. Our study revealed that vitamin C levels are undetectable in more than 90% of the patients included. The mechanisms of this significant reduction in vitamin C are uncertain. We hypothesized that several mechanisms, such as increased metabolic consumption due to the enhanced inflammatory response, glomerular hyperfiltration, dialysis, decreased gastrointestinal absorption, or decreased recycling of dehydroascorbate to ascorbic acid, may be involved.

Moreover, vitamin C may have implications for treatment of COVID-19-associated ARDS [3]. Indeed, one preclinical study showed that vitamin C increased resistance to infection caused by coronavirus [4]. Moreover, other clinical studies that included surgical patients and patients with pneumonia showed encouraging results in terms of decreased incidence and severity of lung injury and mortality [5].

Our study has several limitations mainly related with the fact that it is a unicentric study with small sample size and blood sample was obtained in different days of their course in the ICU.

In conclusion, in our cohort of patients with COVID-19-associated ARDS, the levels of vitamin C are extremely low. Despite the limited generalization of these results, we think these findings might stimulate clinicians to measure vitamin C levels in COVID-19 patients to describe the real impact of this alteration.

## Data Availability

The datasets used and/or analyzed during the current study are available from the corresponding author on reasonable request.
